# Natural history of Platypria (Platypria) hystrix (Fabricius, 1798) on Fabaceae host plants, with notes on other *Platypria* species in India (Chrysomelidae, Cassidinae, Hispini)

**DOI:** 10.3897/zookeys.1031.60129

**Published:** 2021-04-14

**Authors:** Sachin Ranade, Kaniyarikkal Divakaran Prathapan, Hemant V. Ghate, Caroline S. Chaboo

**Affiliations:** 1 Bombay Natural History Society, Vulture Conservation Breeding Center, Rani – 781131, Kamrup District, Assam, India Bombay Natural History Society Rani India; 2 Department of Entomology, Kerala Agricultural University, Vellayani P. O., Trivandrum – 695 522, Kerala, India Kerala Agricultural University Kerala India; 3 Post-Graduate Research Centre, Department of Zoology, Modern College of Arts, Science and Commerce, Shivajinagar, Pune 411 005, India Modern College of Arts, Science and Commerce Pune India; 4 University of Nebraska State Museum, Systematics Research Collections, W436 Nebraska Hall, University of Nebraska, Lincoln, Nebraska 68588-0514, USA. University of Nebraska State Museum Lincoln United States of America

**Keywords:** Leaf miner, life history, hispine, *Erythrina*, *Gouania*, *Mucuna*, *Pueraria*, *Ziziphus*

## Abstract

The leaf-beetle genus *Platypria* Guérin-Méneville, 1840 comprises two subgenera and 34 species (Chrysomelidae: Cassidinae: Hispini). Host plants are documented for eight species and indicate mostly perennial species of Fabaceae and Rhamnaceae. Larvae and pupae have been documented for two *Platypria* species. This paper presents novel natural history data, based on a field study of populations of Platypria (Platypria) hystrix (Fabricius, 1798) on *Erythrinastricta* Roxb. and *Puerariaphaseoloides* (Roxb.) Benth. in Kerala, south India and on *Erythrinavariegata* L., Puerariamontanavar.lobata (Willd.) Maes. & S. Almeida and *Mucunapruriens* (L) DC in Assam, northeast India. Three new Fabaceae hosts are reported for P. (P.) hystrix. Brief notes and new host records, based on field observations, are also provided for the other three species of *Platypria* in India – P. (P.) chiroptera Gestro, 1899, P. (P.) echidna Guérin-Méneville, 1840 and P. (P.) erinaceus (Fabricius, 1801). *Platypria* females slit the leaf to lay a single egg which is covered with secretions that harden as an ootheca, the egg covering in Cassidinae*s. l.* There are five larval stages, each with the typical ‘hispine’ mining form and behaviour – a flattened cream-coloured body, chitinised head capsule and claws, and feeding on mesophyll and leaving irregular blotch mines on the host leaves. Pupation occurs in an independent pupal mine and lasts about a week. These observations suggest new potential phylogenetic character hypotheses that can stimulate better data collection on leaf-mining Cassidinae and help resolve evolutionary patterns amongst these basal mining genera.

## Introduction

The Old World cassidine tribe Hispini Gyllenhal, 1813 (Coleoptera: Chrysomelidae: Cassidinae) currently comprises 25 genera and 627 species, including three fossil species (Staines 2015). Tribal monophyly is well-supported by the distinct long stiff spines on the pronotum and elytra (Würmli 1975; Chen et al. 1986), particularly the spinose lateral elytral edges (Chaboo 2007:179).

**Figure 1.**
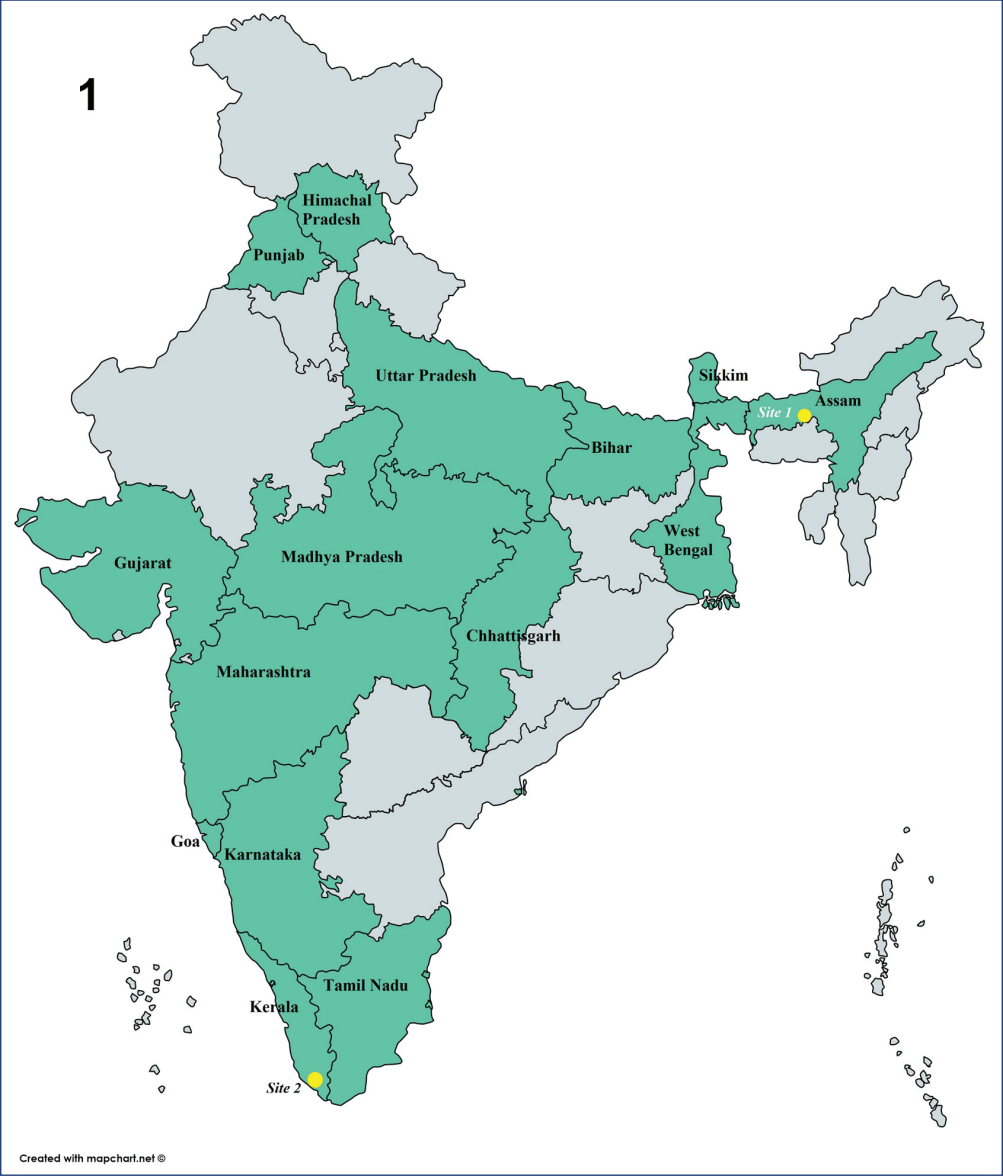
Map showing distribution of *Platypria* in India by state (in green) and our two field sites (yellow circles).

**Figures 2–5.**
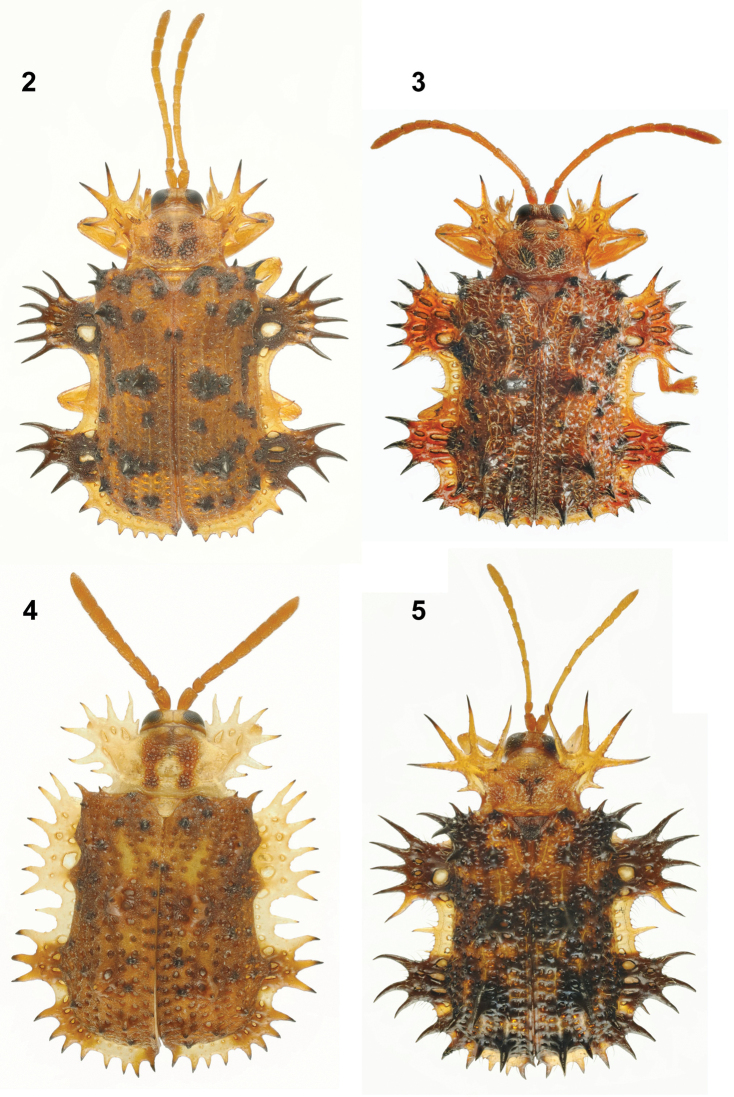
*Platypria* species in India (photos: K.D. Prathapan) **2**Platypria (Platypria) chiroptera Gestro, 1899 **3**Platypria (Platypria) echidna Guérin-Méneville, 1840 **4**Platypria (Platypria) erinaceus (Fabricius, 1801) **5**Platypria (Platypria) hystrix Maulik, 1919.

The genus *Platypria* Guérin-Méneville, 1840 comprises two subgenera (*Platypria*, *Dichirispa*) and 34 species (Staines 2015). The two subgenera are separated by the elytra margins expanded both at the humeri and posteriorly, with long spines and with “windows” in the nominotypical subgenus (fenestrate; Würmli 1975, 1978). This paper focuses on four species found in India (Fig. 1). Adults of Indian *Platypria* are morphologically distinct (Figs 2–5): the body is oblong, but the margins of the pronotum and elytra are expanded into broad rounded lobes and have prominent spinose extensions (Maulik 1919; Uhmann 1954b; Würmli 1975). The antenna has nine antennomeres, as the last three are apparently fused (Maulik 1919). *Platypria* is distributed across the Afrotropical and Oriental Regions. Hosts are known for eight of the 34 species in the genus (Table 1). Kalshoven (1957) noted that *Platypria* is amongst a few Oriental hispine genera atypically associated with eudicotyledonous plants, often belonging to unrelated families; other such genera are *Notosacantha* Chevrolat, 1837 (Rane et al. 2000), *Oncocephala* Agassiz 1846 (Calcetas et al. 2020), *Dactylispa*, *Dicladispa*, *Hispa* and *Monohispa* (Staines 2015).

Juveniles were briefly noted for P. (P.) erinaceus (Fabricius, 1801) (= P. (P.) andrewesi in Beeson 1941, Uhmann 1957, 1958b), P. (Dichirispa) coronata (Guérin-Méneville, 1840) (Uhmann 1958a) and P. (P.) melli Uhmann, 1954a (Chen 1982). Larvae and pupae of P. (P.) melli were further studied on *Hoveniaacerba* Lindl. (Rhamnaceae) by Liao et al. (2014). *Platypria* species have been reported as pests of pear and plum (Chen 1982; Qi et al. 1995) and soybean in China (Kezhen 1992) and as minor pests of trees and shrubs of Fabaceae and Rhamnaceae (Kalshoven 1957). The pest status has been confirmed by others (Ayyar 1940; Mathur and Singh 1959; Nair 1986; Balikai 1999; Rani and Sridhar 2004; Liu et al. 2019).

Four species of *Platypria* have been documented in India (Staines 2015): P. (P.) chiroptera Gestro, 1899 (Fig. 2), P. (P.) echidna Guérin-Méneville, 1840 (Fig. 3), P. (P.) erinaceus (Fabricius, 1801) (Fig. 4) and P. (P.) hystrix Maulik, 1919 (Fig. 5). Maulik (1919) indicated seven species, but some have since been synonymised. Würmli (1975) recorded P. (P.) fenestrata Pic, 1924, which occurs in China and Vietnam, from the Nilgiri Hills in south India; however, this was questioned by Kimoto (1999) who suggested that it could be *P.parva* Chen & Sun, 1964, which occurs in China and Vietnam. Staines (2015) cited P. (P.) fenestrata as a fifth species for India. However, we never encountered this species in India, despite extensive fieldwork in the country.

We present the first natural history notes on P. (P.) hystrix from two widely-separated localities in India. This species is widespread in southeast Asia and is documented from 16 States in India – Assam, Bihar, Chhattisgarh, Goa, Gujarat, Himachal Pradesh, Karnataka, Kerala, Madhya Pradesh, Maharashtra, Pondicherry, Punjab, Sikkim, Tamil Nadu, Uttar Pradesh and West Bengal (Fig. 1) (Maulik 1919; Basu 1999; Borowiec and Świętojańska 2007; Borowiec and Sekerka 2010; Staines 2015). At present, 20 host plants in six families have been recorded for this species (Table 1). We report observations of the species on four host plants, three being new records for this species. We also present brief biological notes for the other three Indian *Platypria* species based on field observations and provide a key to identify these four species in India. This paper is the first step in an ongoing process; a detailed comparative morphology study is our next goal.

## Materials and methods

The study is based on independent observations by SR and KDP of live populations of P. (P.) hystrix at two sites in India, 2,500 km apart (Fig. 1). Authors SR, KDP and HVG started observations independently and now are collaborating; we pool data here in this phase 1 of a long-term study.

**Figures 6–9.**
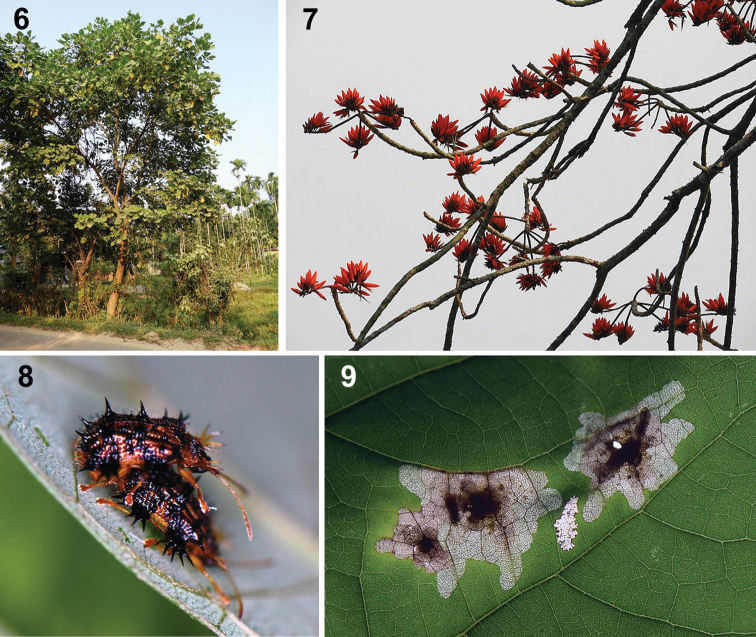
Natural history of Platypria (Platypria) hystrix on *Erythrinavariegata* L., Assam, India **6** host tree **7** flowers **8** adults in copula **9** larval mines. (Photos: S. Ranade).

*Site 1*: INDIA: Assam, Kamrup District, 26°0'0.9"N, 91°32'53.7936"E, 190 m elev., September 2019–May 2020 (Figs 6–9). Beetles were observed by SR on a single tree, *Erythrinavariegata* L. (Fabaceae) that was visited frequently to record natural history data. Specimens were not collected at that time, but photographs and movies with a SLR camera were recorded. Populations were also observed on two perennial vines *Mucunapruriens* (L.) DC and Puerariamontanavar.lobata (Willd.) Maes. & S. Almeida (Fabaceae) at the same location, October–November 2020. We are continuing with the natural history study of this population.

**Figures 10–13.**
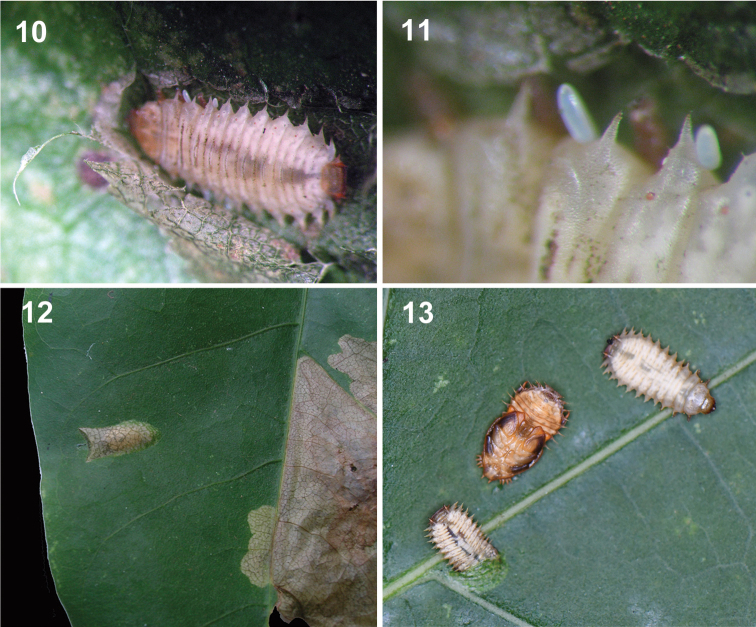
Juveniles of Platypria (Platypria) hystrix on *Erythrina* L. spp. in India **10** Larva **11** Eggs of parasitoid wasp on larva. (Photos: K.D. Prathapan in Kerala; on *E.stricta* Roxb.) **12** Pupal chamber **13** Pupae and a last instar larva initiating pupal mine (Photo: S. Ranade in Assam; on *E.variegata* L.).

**Figures 14–21.**
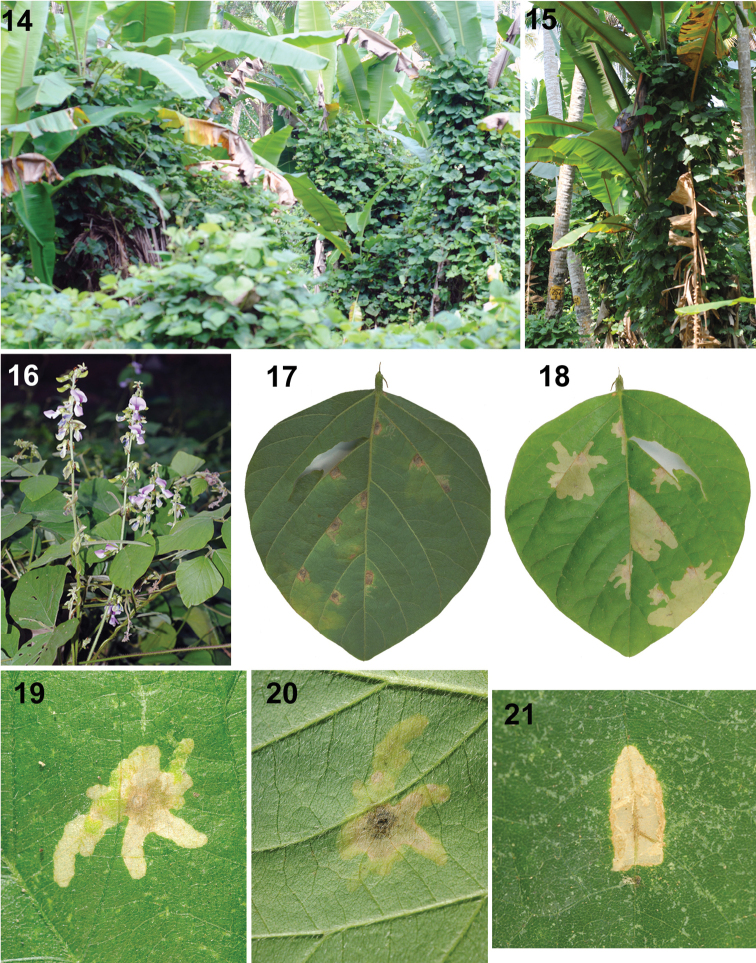
Natural history of Platypria (Platypria) hystrix on *Puerariaphaseoloides*, Kerala, India (Photos: K.D. Prathapan) **14** Vine growing over all plants in a banana plantation **15** Plant growing over banana **16** Inflorescence **17** Leaf with six larval mines, abaxial view **18** Leaf with six larval mines, adaxial view **19** Larval mine, view from adaxial side of the leaf **20** Larval mine, view from abaxial side of the leaf with ootheca at the centre **21** Pupal chamber.

*Site 2*: INDIA: Kerala, Vellayani, Kerala Agricultural University campus, 8°25'46.3"N, 76°59'07.8"E, 39 m elev. Author KDP observed this population for ca. three months in 2007 and again from October 2019 to May 2020. During 2007, populations were observed on *Erythrinastricta* Roxb. (Figs 10–13) and *Puerariaphaseoloides* (Roxb.) Benth. (Figs 14–21) (both Fabaceae). However, the entire population of *Erythrina* was decimated following the invasion of the *Erythrina* gall wasp, *Quadrastichuserythrinae* Kim (Hymenoptera: Eulophidae) (Faizal et al. 2006). In 2019 and 2020, the beetles were found only on *P.phaseoloides* at Vellayani (Figs 14–21).

### Rearing

We marked and numbered leaves with larval mines to observe their behaviour and development. In Assam, we followed 15 larvae and four successfully reached adulthood. In Kerala, about 20 larval and pupal mines were studied. Some specimens were taken to the lab to rear and collect certain life stages for vouchers, photography and measurements.

In addition to the detailed study of P. (P.) hystrix above, HVG, PKD and SR observed and collected the other three Indian *Platypria* species on *Ziziphus* and other hosts in India and provide these brief notes below.

#### Natural history notes on Platypria (Platypria) chiroptera Gestro, 1899

PKD and M. K. Shameem collected this species in six localities in the southern Western Ghats, India: Karnataka, Kalasa, 11.V.2011, Shameem K. (2 specimens); Kottigehara, 22.IX.2004, Prathapan Coll. (1 specimen); Kottigehara, 13°7'7.7"N/ 75°30'7.9"E, 938 m a.s.l., 8.v.2011, Prathapan and Shameem (2 specimens); Kerala, Neyyar W. L. San., 8.II.2002, Prathapan Coll. (1 specimen); Elappara, 1.III.2011, Shameem K. (1 specimen); Kuttiyadi, Janakikkadu, 14.iii.2013 (1 specimen); Silent Valley Nat. Park, Sairandhri, 11°5'35.8"N/ 76°26'47.7"E, 1030 m a.s.l., 15.xi.2013, Prathapan and Shameem (4 specimens, KAU). The host plant is *Gouaniamicrocarpa* DC. (Rhamnaceae) (M.K. Shameem, personal communication). Bhasin (1942) and Mathur and Singh (1961) recorded P. (P.) chiroptera (as *Platypriagarthwaitei* Bhasin, 1942) on *Ziziphusincurva* (Rhamnaceae).

#### Natural history notes for Platypria (Platypria) echidna Guérin-Méneville, 1840 (Figs 26–28)

Authors HVG and SR observed live populations on four different hosts, all new records, in India. Locality 1: Pune District, Tamhini-Dongarwadi, Mulshi, 18°26'48.1488"N, 73°25'29.3808"E, June–September (monsoon season) 1997–2001. Locality 2: Pune, Paud Road, 18°30'24.066"N, 73°46'58.2708"E, 10 April 2011. These adults were noted feeding by scraping the upper leaf surface, *Ziziphusrugosa* Lam. Localities 3–4: Adults were observed feeding on *Z.nummularia* and on *Z.mauritiana* Lam. at Pune District, Bhimashankar, 19°4'36.1848"N, 73°32'6.8784"E, August 1999 and on *Ziziphusxylopyrus* (Retz.) Willd., Pashan, Pune, 18°32'12.1884"N, 73°47'22.6284"E, May 1999. Locality 5: Larvae, pupae, and adults together were observed only on *Z.mauritiana*, at Taljai Tekadi Pune, 18°31'13.548"N, 73°51'24.2784"E, September 2007.

**Figures 22–28.**
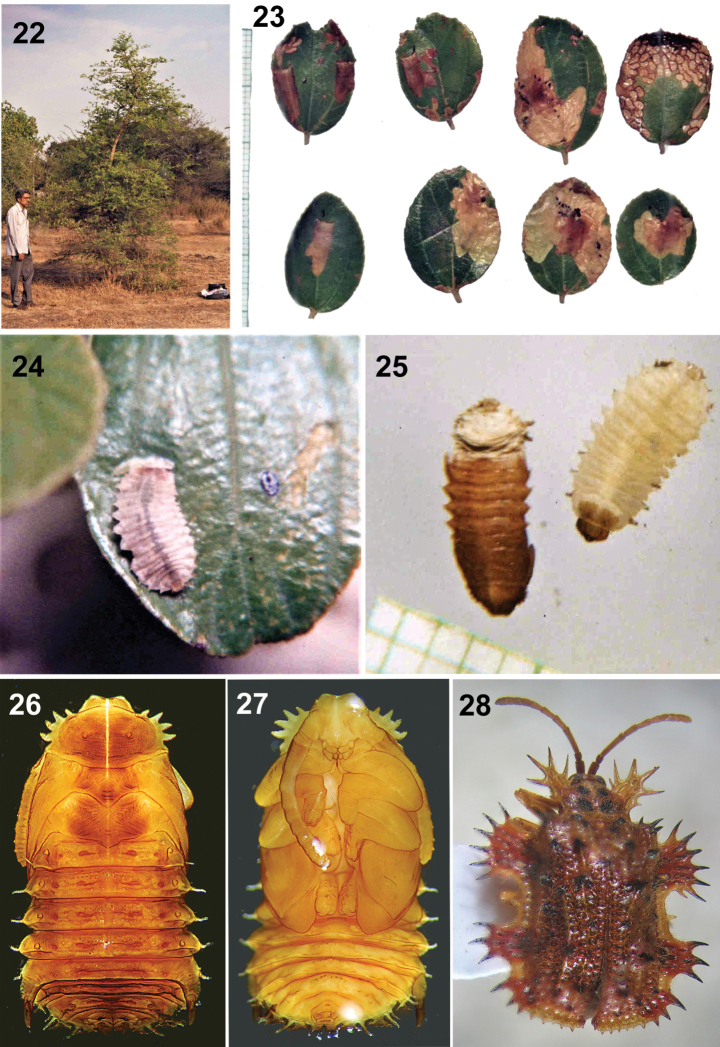
**22–25** Natural history of Platypria (Platypria) erinaceus on *Ziziphusnummularia* (Burm.f.) Wight & Arn., Maharashtra, India, (Photos: S. Ranade) **22***Ziziphusxylopyrus* (with author HVG standing) **23** larval mines in small, rounded leaves of *Ziziphusnummularia***24** larva **25** mature larva and pupa **26–28**Platypria (Platypria) echidna**26** Pupa, dorsal view **27** pupa, ventral view **28** adult, dorsal view. (Photos: H.V. Ghate).

#### Natural history notes for Platypria (Platypria) erinaceus (Fabricius, 1801)

HVG and SR observed this species on the host plant, *Z.mauritiana* Lam. (Formerly *Z.jujuba* Lamk.) in several sites in India. Locality 1: Pune District, Chatushrungi, 18°32'12.4872"N, 73°49'42.69"E, 27 May 1999. Locality 2: Pashan, 18°32'12.1884"N, 73°47'22.6284"E, 27 May 1999. Locality 3: West Bengal, Kolkata, Baruipur 22°22.770"N, 88°26.154"E, 9 m a.s.l., 19.vi.2013, KDP Coll. (3, KAU). Locality 4: Tamil Nadu, Manavur, 13°05'48.44"N, 79°47'37.66"E, 54 m a.s.l., Ex. *Ziziphus*, 24.ix.2016, Shameem KM Coll. (1, KAU). Locality 5: Pune, Paud, NDA Road, on *Z.nummularia* (Burm.f.) Wight & Arn. (HVG); adults were feeding by scraping the upper surface of leaf. Mating pairs, larvae and pupae were also noted.

### Taxonomic identifications

*Erythrinastricta* (Fabaceae) was identified by A. K. Pradeep, Calicut University Herbarium, previously for Faizal et al. (2006). No plant voucher was collected at that time as no flowers were produced under Vellayani conditions; now the plant has become locally extinct. *Puerariaphaseoloides* was identified by A. P. Balan, Malabar Botanical Garden. *Erythrinavariegata*, P.montanavar.lobata and *M.pruriens* (all Fabaceae) from Assam were identified by G. Krishna, Central National Herbarium (CAL), Botanical Survey of India. The beetles were identified independently by authors KDP and SR as P. (P.) hystrix using the species key by Maulik (1919) and compared with photos of type specimens deposited in Berlin Museum of Natural History and Kiel University, Germany. A key to identify the four *Platypria* species in India is developed.

### Specimen collections and repository

Specimens collected by KDP and associates over years from various localities in India are deposited in the Travancore Insect Collection, Kerala Agricultural University, Vellayani (KAU). In addition to KAU, specimens of beetles will be deposited also at the National Bureau of Agricultural Insect Resources, Bengaluru, India (NBAIR). Specimens of P. (P.) echidna and P. (P.) erinaceus are deposited at the Modern College of Arts, Science and Commerce, Pune, India. Additionally, a specimen series of P. (P.) hystrix is on loan from KDP to CSC for further study. Vouchers of *P.phaseoloides* (Accession no. 7019), P.montanavar.lobata (Accession no. 7030, 7031) and *M.pruriens* (Accession no. 7037, 7038) are deposited in the Calicut University Herbarium, Department of Botany, University of Calicut, Kerala.

### Host plant ecology

The four Fabaceae hosts are native to southeast Asia. Each is used for multiple purposes in agro-ecosystems. *Erythrinastricta* is a spinose tree on which cultivated black pepper (*Pipernigrum* L.) is trailed. It is also grown as a hedge plant and shade tree. Leaves are used as fodder for sheep and rabbit (Prathapan, personal observations; Sastri 1952). *Erythrinavariegata* (Fig. 6) is a tropical soft-wood tree, closely resembling *E.stricta*; however, its stem is usually unarmed. It is cultivated as an avenue tree and a live fence and it is used as a shade tree in plantations of tea and coffee and to trail betel vine and black pepper (Sastri 1952; Prathapan, personal observations). *Puerariaphaseoloides* (Figs 14–18; tropical kudzu) is a perennial climbing vine, trailing over trees, shrubs, bananas and grasses in and around the Instructional Farm of Kerala Agricultural University, Vellayani, India. It is grown as a cover crop in rubber plantations and for fodder (see Keung 2002). Puerariamontanavar.lobata, known for rapid and competitive growth, is used as a pasture, fodder and hay crop in North America (Lindgren et al. 2013). *Mucunapruriens* is used for its medicinal properties and as fodder (Choudhary et al. 2012; Patiri and Borah 2007). These five hosts have moderately large, trifoliate leaves.

The genus *Ziziphus* Mill. includes about 58 species of spiny shrubs and trees (El Maaiden et al. 2020). It is extensively used in folk and traditional medicine in arid and semi-arid regions for the treatment of diarrhoea, dysentery, cholera, diabetes, hypertension, inflammation, intestinal spasm, liver, malaria and other diseases (El Maaiden et al. 2020). *Ziziphusmauritiana* Lam., called Indian jujube or ber, is a tropical shrub or small tree, of considerable commercial importance and is widely cultivated for its fruits. *Ziziphusrugosa* Lam., called wild jujube, is a thorny tree or straggling shrub, common in foothills and low mountains in India (Chadha 1976). Fruits are collected from the wild for consumption. *Ziziphusxylopyrus* (Retz.) Willd., locally called ‘kath ber’, is an erect shrub or small tree, common in dry and deciduous forests (Chadha 1976). *Ziziphusnummularia* (Burm.f.) Wight & Arn., occurring in semi-arid areas from Iran to the Indian subcontinent, is a multipurpose branched thorny shrub reaching a height of 1–3 m, with medicinal, nutritional, industrial and economic values (Zandifar et al. 2020).

### Photographs

Specimens were colour-photographed using a AF Micro Nikkor 60 mm macrolens, mounted on a Nikon D3000 SLR camera. The camera was mounted on a Wemacro stack rail, positioned vertically. Three Ikea 201.696.58 Jansjo Desk Work LED Lamps, with suitable diffusers, were used to uniformly illuminate the specimen. A Wemacro rail android Bluetooth control app, installed on a smartphone, was used to remotely control the imaging system. Multiple images at different depths of plane were taken and were stacked together using Helicon focus software. The high-resolution images, thus obtained, were edited with Adobe Photoshop 2020. Field photographs were taken using a Canon EFS 55–250 mm lens mounted on a Canon EOS 1300D SLR camera or Micro Nikkor 60 mm macrolens mounted on a Nikon D3000 SLR camera.

### Measurements

Life stages of P. (P.) hystrix were measured using a standardised ocular micrometer placed in one eyepiece of a stereoscopic microscope. Measurements of host plant leaves and leaf mines were taken using a Vernier caliper. In our Assam lab, we measured three adults, one instar I, one instar III, one instar V, one pupa and one pupal mine. In our Kerala lab, we measured 10 adults, 20 pupal mines, 10 pupae, and seven oothecae.

### Taxonomy

We use the current plant names according to the online catalogue (Tropicos 2020) and current beetle names according to the catalogue of Staines (2015).

## Results

### Key to species of *Platypria* in India

**Table:** 

1	Antenna thick, hardly extending beyond scutellum over pronotum; third antennomere not longer than 2.5 times width	**P. (P.) erinaceus (F.) (Fig. 4)**
–	Antenna thin, extending well beyond scutellum over pronotum; third antennomere about four times as long as wide	**2**
2	Anterior lateral lobe on each side of elytra has five spines; feeds on Fabaceae	**P. (P.) hystrix (F.) (Fig. 5)**
–	Anterior lateral lobe on each side of elytra has six spines; feeds on Rhamnaceae	**3**
3	Elytra covered with white pubescence; punctures large, subquadrate and contiguous; anterior and posterior lateral lobes on elytra reddish	**P. (P.) echidna Guérin-Méneville (Fig. 3)**
–	Elytra glabrous; punctures rounded, separated by broad interstices; anterior and posterior lateral lobes of elytra blackish	**P. (P.) chiroptera Gestro (Fig. 2)**

### Natural history of Platypria (Platypria) hystrix

We report *Erythrinastricta*, Puerariamontanavar.lobata and *Puerariaphaseoloides* as new hosts for P. (P.) hystrix (Figs 6–21). In India, this beetle has been reported on other species in these genera, as well as on species of *Cajanus*, *Desmodium* and *Dolichos* (Table 1, all citations therein).

**Table 1.** Host plants of *Platypria* species (Cassidinae: Hispini). New host records are indicated by bold font and ‘*’.

Species	Host family	Host species	Reference
**Platypria (Platypria) sp.**	Fagaceae	*Quercussemecarpifolia* Sm.	Stebbing 1914
**Platypria (Platypria) chiropteraGestro 1899** (=*Platypriagarthwaitei*Bhasin 1942)	Rhamnaceae	***Gouaniamicrocarpa* DC.**	This paper*
		*Ziziphusincurva* Roxb.	Bhasin 1942; Mathur and Singh 1961
**Platypria (Dichirispa) coronata (Guérin-Méneville 1840)**	Fabaceae	*Desmodiumrepandum* (Vahl) Poir.	Uhmann 1958a
		*Puerariaphaseoloides* (Roxburgh) Bentham	Bernon and Graves 1979
**Platypria (Platypria) echidna Guérin-Méneville 1840**	Euphorbiaceae	*Mallotus* Lour. sp.	Hua 2002
	Fabaceae	*Desmodiumgangeticum* (L.) DC	Beeson 1941
		*Erythrina* L. sp.	Beeson 1941; Ayyar 1940
		*Erythrinasubumbrans* (Hassk.) Merr. (= *Erythrinalithosperma* Blume ex. Miq.)	Fletcher 1921; Chatterjee and Bhasin 1936; Kalshoven 1957; Mathur and Singh 1959; Zaka-Ur-Rab 1991
		*Erythrinavariegata* L. (=*Erythrinaindica* Lam.)	Zaka-Ur-Rab 1991
		*Erythrinavariegataorientalis* Murr.	Hua 2002
		*Puerariatuberosa* (Roxb. ex Willd.) DC.	Beeson 1941
	Rhamnaceae	*Ziziphus* Mill. sp.	Beeson 1941; Mathur and Singh 1961
		***Ziziphusmauritiana* Lam.**	This paper*
		***Ziziphusnummularia* (Burm.f.) Wight & Arn.**	This paper*
		*Ziziphusoenoplia* (L.) Mill.	Chatterjee and Bhasin 1936
		***Ziziphusrugosa* Lam.**	This paper*
		***Ziziphusxylopyrus* (Retz.) Willd.**	This paper*
**Platypria (Platypria) erinaceus (Fabricius 1801b)** (=*Platypriaandrewesi* Weise 1904)	Fabaceae	*Desmodiumgangeticum* (L.) DC.	Beeson 1941
		*Erythrina* L. sp.	Beeson 1941; Kalshoven 1957
		*Puerariatuberosa* (Roxb. ex Willd.) DC.	Beeson 1941
	Poaceae	*Oryzasativa* L.	Anand 1989
		*Saccharum* L. sp. (“sugar-cane”)	Maulik 1919, 1937
	Rhamnaceae	*Ziziphus* Mill. spp.	Maulik 1919, 1937; Chatterjee and Bhasin 1936; Beeson 1941; Mathur and Singh 1961
		*Ziziphusjujuba* Lam. (= *Ziziphusmauritiana* Lam.)	Maxwell-Lefroy 1909; Stebbing 1914; Beeson 1919; Maulik 1919, 1937; Fletcher 1921; Ayyar 1940; Speyer 1954; Kalshoven 1957; Mathur and Singh 1961; Nair 1986; Zaka-Ur-Rab 1991; Balikai 1999; Kalaichelvan and Verma 2005
		***Ziziphusnummularia* (Burm.f.) Wight & Arn.**	**This paper**
**Platypria (Platypria) hystrix (Fabricius 1798)**	Fabaceae	*Cajanuscajan* (L.) Millsp.	Uhmann 1954a
		*Cajanusindicus* Spreng.	Kalshoven 1957
		*Desmodiumgangeticum* (L.) DC	Beeson 1941
		*Dolichoslablab* L.	Fletcher 1921; Ayyar 1940; Kalshoven 1957; Nair 1986; Zaka-Ur-Rab 1991
		*Erythrina* L. sp.	Fletcher 1914, 1921; Ayyar 1940; Beeson 1941; Kalshoven 1951, 1957
		*Erythrinaarborescens* Roxb. (swarming only)	Chatterjee and Bhasin 1936; Kalshoven 1957
		***Erythrinastricta* Roxb.**	This paper*
		*Erythrinasubumbrans* (Hassk.) Merr. (=*Erythrinalithosperma* Blume ex Miq.)	Zaka-Ur-Rab 1991
		*Erythrinavariegata* L. (= *Erythrinaindica* Zoll.)	Maulik 1919, 1937; Beeson 1919; Chatterjee and Bhasin 1936; Gressitt and Kimoto 1963; Gressitt 1950; Kalshoven 1951; Speyer 1954; Mathur and Singh 1959; Zaka-Ur-Rab 1991
**Platypria (Platypria) hystrix (Fabricius 1798)**	Fabaceae	*Erythrinavariegataorientalis* Murr.	Hua 2002
		*Glycinemax* (L.) Merr. (“soybean”)	Kezhen 1992
		*Mucunapruriens* (L.) DC	Rani and Sridhar 2004
		*Phaseolus* spp.	Kezhen 1992
		**Puerariamontanavar.lobata (Willd.) Maes. & S. Almeida**	This paper*
		***Puerariaphaseoloides* (Roxb.) Benth.**	This paper*
		*Puerariatuberosa* (Roxb. ex Willd.) DC.	Beeson 1941
		*Sesbania* Scop. sp. (“agathi”)	Fletcher 1921; Kalshoven 1957; Nair 1986
		*Sesbaniaaculeata* (Schreb.) Poir.	Zaka-Ur-Rab 1991; Hua 2002
		*Sesbaniagrandiflora* (L.) Poir.	Beeson 1919; Chatterjee and Bhasin 1936; Speyer 1954; Kalshoven 1957; Zaka-Ur-Rab 1991
		*Sesbania* Scop. sp. (“agathi”)	Fletcher 1921; Chatterjee and Bhasin 1936; Nair 1986
		*Tephrosiacandida* DC.	Kalshoven 1951; 1957
	Fagaceae	*Castanea* Mill. sp. (“chestnut”)	Nair 1986
	Myricaceae	*Myrica* L. sp. (swarming only)	Chatterjee and Bhasin 1936; Kalshoven 1957
		*Myricarubra* (Lour.) Siebold & Zucc.	Hua 2002
	Rhamnaceae	*Ziziphus* Mill. spp.	Beeson 1941; Mathur and Singh 1961
	Rosaceae	*Rubus* L. *sp.*	Hua 2002
		*Rubusellipticus* Sm. (swarming only)	Chatterjee and Bhasin 1936; Kalshoven 1957
	Rubiaceae	*Uncariagambir* (W. Hunter) Roxb.	Kalshoven 1957
**Platypria (Platypria) melli Uhmann 1954**	Poaceae	*Oryzasativa* L.	Gressitt and Kimoto 1963
	Rhamnaceae	*Hovenia*acerba Lindl.	Chen et al. 1986; Liao et al. 2014; Liu et al. 2019
		*Paliurusramosissimus* Poir.	Chen 1982; Hua 2002
		*Ziziphusjujuba* Lam.	Chen et al. 1986; Hua 2002; Liu et al. 2019
**Platypria (Dichirispa) paucispinosa Gestro 1904**	Icacinaceae	*Icacinamannii* Oliv.	Uhmann 1954
***Platypria* sp.**	Rosaceae	*Pyrus* sp. (“pear”)	Qi et al. 1995
		*Prunus* sp. (“plum”)	Qi et al. 1995

Starting on 17 September 2019, SR observed irregular blotch mining on leaves of a young tree of *E.variegata* (Fig. 9). Eggs were observed on *E.variegata* in Assam as well as on *P.phaseoloides* in Kerala. They were laid singly on the adaxial side of leaves. Up to four eggs were observed on a single leaflet. Individual eggs were inserted into a depression made on the mesophyll and were covered with a creamy-brown secretion to form the oblong-oval ootheca, that measured 1.03–1.32 mm (1.10 mm – mean of 7 observations) long and 1.07–1.48 times (1.24 times – mean of 7 observations) longer than wide. A characteristic, long, thread-like process, arising from the middle of the ootheca, enabled easy identification of the ootheca under low power of the microscope. From the abaxial side, the ootheca appeared like a minute, brown speck. The egg appeared soft and was easily ruptured when we attempted to separate it from the oothecal covering. The thread-like process and the outer wall of the ootheca remained intact even after hatching and formation of the leaf mine. In Assam, a female was observed on the host plant for ten days; oviposition and egg hatch were noted. Twenty-one leaflets were observed, each with about 3–4 beetle eggs. These eggs hatched in about 4–7 days. Many eggs remained unhatched or the larvae died prematurely.

The larva hatched out of the egg mines into the adjacent mesophyll without breaking the oothecal covering. It feeds and moves within the leaf creating mines by consuming mesophyll tissue. The first instar larva grew up to 1.8 mm. The larva has chitinous brown head and translucent-greenish body. The alimentary canal appears dark green due to the presence of food. While observing it against sunlight, the mines appeared occupied and small larvae were apparent through the epidermis. The larval mine in *P.phaseoloides* appeared less apparent in the abaxial view (Fig. 17), but clear and rather transparent from the adaxial side of the leaf lamina (Fig. 18). The larval mines are irregular blotch mines. Six leaf mines were observed on a 65 mm wide leaflet of *P.phaseoloides* (Figs 17–18) at Vellayani. The mines contained excreta, exuviae and often remnants of dead larvae. The leaf mines of the late instars were noticeable as some of them were approximately 1 cm wide and 10 cm long and irregularly shaped. The final instar was about 5 mm long.

#### Pupation

The pupation takes place in a separate pupal mine. Emergence from the larval mine and construction of the pupal mine were observed in Assam. The mature larva (Fig. 13) exits the larval mine, moves towards the other leaf end and initiates the pupal mine. Construction of the pupal mine by a single larva that was observed took 23 minutes to conceal itself. Four pupal mines were observed in Assam on *E.variegata*. The average size of the pupal mine was 9 × 4 mm (n = 4). Excreta was present next to every pupal mine’s single opening. The pupal period in Assam lasted for about seven days. On four occasions, the pupa was observed moving out of the mine and adults emerged in early morning.

About 20 pupal mines were observed on *P.phaseoloides* at Vellayani. The length of pupal mines ranged from 7.5–10.1 mm (9.98 mm; mean of 10 observations) and width 3.5–4.5 mm (4.01; mean of 10 observations). All, except two, were formed along a leaf vein. Two were formed between the veins on the leaf lamina. The pupal mines are U-shaped, resembling a pocket, with its distal end closed and the proximal end, from where the larva initiated the mine, remaining open. The resident pupa has the head orientated to the closed end and its rear end towards the mine opening. In Assam, we observed that a pupating larva spent one day in the pupal mine, then cast the last larval skin and pupated. This individual took 9 days from formation of the pupal mine to adult emergence. Generally, 1–2 pupal mines were observed on a single leaflet on both *E.variegata* in Assam and *P.phaseoloides* in south India (Vellayani, Kerala). The fresh pupa is yellow in colour that turns coffee-brown in a few days. Prior to the emergence of the adult, the pupa exited the mine and shed the exuviae. In the case of the single individual observed by PKD in the laboratory, the exuviae of the pupa remained about 3 cm away from the pupal mine. Thus, the pupa can move out of the mine to eclose.

The adults (n = 10; length 4.29–5.24 mm) were observed feeding mainly by scraping on adaxial surface of leaves. Sexual dimorphism was not distinct to the naked eye. Copulation was recorded in the morning as well as in the evening. Pairs were in copula for more than an hour.

#### Dormancy and aestivation

In Assam, the adults were seen until the first week of December 2019, after which they were not found anymore. They appeared on the same plant in the first week of March 2020. Further south, at Vellayani in Kerala, the population of P. (P.) hystrix on *P.phaseoloides* was active throughout the year, as adults and leaf mines were observed even during the summer months of March and April. Apparently, no dormant stage of the insect occurs in Kerala as extremes of climate are absent in this part of the country.

#### Longevity

Although our observations are still in progress, we noticed that adults emerging in September 2019 in Assam were active, with mating and egg laying observed during March 2020. We suspect that the adults survive for at least one year.

#### Mating behaviour

Copulation was observed in the third week of March after several thunder showers in Assam. On 23 March, we noted four pairs on *E.variegata*. In the case of two pairs, a single female was pursued by two males. The male mounted the female, keeping fore- and middle legs on the elytra of the female, the hind legs being on the substratum. The pair remained coupled for more than one hour per observation. On a few occasions, coupled pairs were observed for 4–6 hours. The female moved around, carrying the male and even fed while in copula. During a single sighting, we found a maximum of eight beetles on a single sapling of *Erythrina* at Assam, indicating that it is not a major pest.

#### Natural enemies

At the Vellayani site, we observed a Braconidae wasp (Hymenoptera) parasitising a mature larva of P. (P.) hystrix (Fig. 11) and ant (Hymenoptera: Formicidae) predation of a pupa. Both the wasp and ant specimens are deposited at KAU. In the Pune locality, we observed a chalcid wasp (Hymenoptera) laying its egg on a late larval instar on 27 May 1999 and subsequently, we detected a chalcid infestation of the larval and pupal stages of P. (P.) erinaceus. Bernon and Graves (1979) is the only other report of Hymenoptera parasites of *Platypria*; they noted that *Platypria* was an alternative host of the Hymenoptera parasites of the *Coelaenomenodera* pest.

## Discussion

Santiago-Blay (2004) has discussed many aspects of leaf mining by Chrysomelidae and Chaboo (2007: 46–47) provides an overview of Cassidinae pupation. We discuss here aspects of the biology and behaviour of *Platypria* species and compare with the other members of the tribe and Cassidinae*s. l.* generally. We discuss refinements for the current morphology and behaviour-based phylogenetic characters of Chaboo (2007).

### Plant relations

*Platypria* is associated mainly with two plant families, Fabaceae and Rhamnaceae (Table 1). We found several citations in Indian literature about the genus that should be added to the online catalogue of Staines (2015). Records on other plants – Fagaceae [Nair (1986), Euphorbiaceae (Hua 2002) and Poaceae (Anand 1989; Maulik 1919, 1937)] – need confirmation as there is little information on immatures from these observations. We can call only those plants as ‘hosts’ where larval development occurs successfully. In that sense, P. (P.) echidna may sometimes feed on *Z.nummularia*, but we have never observed larvae or pupa of this species on *Z.nummularia*. Similarly, we never observed larvae/pupae of any *Platypria* species on *Z.xylopyrus* (Retz.) Willd. which we [HVG and SR] regularly visited to study bionomics of another cassidine. Further, although *Z.oenoplia* (L.) Mill. is reported as a host of one *Platypria* species (see Table 1), HVG never observed *Z.oenoplia* in Pune harbouring any *Platypria*.

Kalshoven (1957) noted that *Platypria* is one amongst a few unusual Oriental hispine genera associated with dicotyledonous plants, often belonging to different families. He also commented that *Platypria* is unusual as it is one of the few hispine taxa specific to dicots and exhibits trophic selections between unrelated host plant families.

### Pest status

Ayyar (1940) recorded P. (P.) hystrix as a leaf-feeding pest on *Dolichoslablab*, *Sesbania* sp. and *Erythrina* sp. in south India. He also noted P. (P.) echidna on *Erythrina* sp. in south India and P. (P.) erinaceus on *Z.jujuba*. Nair (1986) recorded P. (P.) erinaceus as a pest on *Z.jujuba*, as well as P. (P.) hystrix on *D.lablab*, *Sesbania* sp. and *Castanea* sp. (Fagaceae, chestnut) in India. Rani and Sridhar (2004) recorded P. (P.) hystrix as a pest damaging leaves of MucunapruriensL. (DC)var.utilis in south India (this plant is used as a nerve tonic and aphrodisiac in Indian traditional medicine). However, P. (P.) melli is known as a significant pest of Rhamnaceae fruit trees, *Hoveniaacerba* and *Ziziphusjujuba*, in China (Liu et al. 2019). In India, there have been no reports of outbreaks or severe crop damage.

### Life cycle

All life stages of P. (P.) hystrix (egg to adults) were observed in both south and northeast India. The natural history of the populations observed in Assam, northeast India and in Kerala, south India were rather identical, irrespective of the host species, though the populations are separated by a distance of > 2,500 km and climates are distinct. The south Indian population at Vellayani was active throughout the year as harsh winter or summer is absent here, while the northeast Indian population vanished as the winter peaked and re-appeared only after receipt of rains in summer, thus disappearing for at least three months from December to March.

Information is limited on the eggs and associated maternal behaviour for leaf-mining hispines. In P. (P.) hystrix, we observed females excavating a depression on the abaxial surface of leaves and laying a single egg there. Then she covered the egg with a yellow secretion that turned red brown on drying and formed a crusty oothecal covering. Thrusting single eggs into the leaf lamina is known in some leaf-mining hispines (Chen 1982; Chaboo et al. 2010; Shameem et al. 2016; Liao et al. 2018b), although Taylor (1937) noted that females of *Promecotheca* species may oviposit on the leaf surface or sink the egg into the leaf and the natal larva starts the mine. In *Prionispachampaka* Maulik, 1919 (Oncocephalini), the female oviposits 5–6 eggs into a channel she cuts on the leaf (Liao et al. 2018a). Chaboo (2007: 244) proposed two egg features (egg stalk and faecal cover) for phylogeny reconstruction; our data here suggest at least three new potential character hypotheses about the oviposition site (externally on leaf surface or thrust into the leaf tissue), egg grouping (single or massed) and maternal covering (naked with no covering, oothecal secretion, faecal/plant covering or oothecal secretion + faecal/plant covering). Verma and Kalaichelvan (2004) reported observations on oothecal structures in Indian Cassidinae; however, our observations of such secretions in *Platypria* indicate the behaviour of maternal coverings is more widespread across the cassidine tree of life. It is very important to document such information in fine detail to achieve better resolved phylogenies of Cassidinae*s. l.*

We observed all larvae of the four Indian species of *Platypria* making a blotch mine, as in some other mining Cassidinae (Bernon and Graves 1979; Chen 1982; Lee et al. 2009; Liao et al. 2014, 2018a). Figs 17–18 show six mines in one leaf; however, we are uncertain how many larvae can be sustained by the single leaflet to reach pupation and adulthood. We observed a single larva per mine, agreeing with observations in *Javetapallida* Baly, 1858 (Shameem et al. 2016) and *Chaeridionathailandica* Kimoto, 1998 (Świętojańska and Kovac 2007). This contrasts with those mining species whose larvae live gregariously in a common mine (e.g. *Pr.champaka*, Liao et al. 2018a).

In our *Platypria* species, the mature larva exits the larval mine and constructs a separate leaf mine for pupation (Figs 12, 13 and 21; see Suppl. material 1 on Pensoft’s Youtube channel: https://www.youtube.com/channel/UC3mfJg-mxTVrXOjE3XrkAMw). This is different from P. (P.) melli Uhmann, 1954 (Liao et al. 2014) where the larva mines into the mid-rib to pupate; such a mid-rib pupation mine is also known in *C.thailandica* (Oncocephalini; Świętojańska and Kovac 2007). The behaviour of a different pupation mine within the leaf blade is also known in some mining cassidines – *Cassidisparelicta* Medvedev, 1957 (Hispini; Liao et al. 2018b), *Oncocephalapromontorii* Péringuey, 1898 (Oncocephalini; Chaboo et al. 2010), *Notosacanthavicaria* (Spaeth, 1913) (Notosacanthini; Rane et al. 2000) and *Pr.champaka* (Liao et al. 2018a). In contrast, other leaf-mining cassidines pupate within the larval mine. Species of *Dactylispa* Weise (Hispini), which feed on either monocots or dicots, pupate within the larval leaf mine (Zaitsev 2012). The rice pest, *Dicladispaarmigera* (Olivier, 1808) (Hispini) and the palm-feeder, *Javetapallida* (Shameem et al. 2016), both have pupation within the larval mine.

The structure of the pupal mine appeared very similar in our observed *Platypria* species. In *C.thailandica* (Oncocephalini), the mature larva exits the larval mine, bores into the mid-rib forming a pupal chamber and then pupates with the head orientated towards the stem of the plant (Świętojańska and Kovac 2007). Members of Hispini, Notosacanthini and Oncocephalini, that live on eudicots, create more or less similar pupal mines.

Chaboo (2007: 244) proposed Character 18 with four states for different pupation sites across Cassidinae*s. l.* Our new observations here suggest that the origin of the pupation mine can provide an additional character hypothesis with two states – within a larval mine or a separate mine.

The pupal mines of P. (P.) hystrix are U-shaped and the resident pupa is positioned such that its rear end is orientated to the single opening at the wider end. This facilitates respiration with the erect, tubular spiracles. Even in rains when the pupal mine may become flooded, the pupa can be seen projecting spiracles out of the opening; the pupa is motile and not glued like other Cassidinae. Similar pupal mines have been reported for P. (P.) echidna and P. (P.) erinaceus and some other basal Cassidinae, such as *Chaeridionapicea* Baly (personal observations SR; Oncocephalini), *Notoscantha* (Rane et al. 2000; Notosacanthini), and *Oncocephalatuberculata* Olivier,1792 (Oncocephalini). Notosacanthini is one of the historic transitional tribes between crown-clade Cassidinae, based on adult morphology and basal “hispines” (Chaboo 2007). The similarity of its pupal chamber to that of *Platypria* and Oncocephalini underscores the need for re-assessment of its taxonomic placement.

Chatterjee and Bhasin (1936) and Kalshoven (1957) reported *Platypria* adults as exhibiting swarming behaviour on *Rubusellipticus* Sm. (Rosaceae) in India. We did not observe such behaviour. Swarming has been reported for only one other Cassidinae, *Caelaenomenoderaelaeidis* Maulik (Bernon and Graves 1979), where this behaviour appears to be cyclical. It could provide another set of phylogenetically informative characters.

*Platypria* females attract many males in a mating frenzy. Once a male is chosen, copulation lasts several hours. (See our supplementary movie file on the life cycle of *Platypria* in India).

## Conclusions

This paper provides a first step in ongoing fieldwork and study of the four Indian species of *Platypria*. We discovered new hosts and note the specialisation of these species on Fabaceae and Rhamnaceae. We characterise aspects of the oviposition behaviour, egg, larvae, pupae, mining behaviour and adult courtship. A detailed morphological study is our next goal.

## Supplementary materials

Supplementary material 1 Movie S1 Data type multimedia Explanation note Natural History of <em full-name="Platypria">Platypria</italic> (<em full-name="Platypria">P.</italic>) <em full-name="hystrix">hystrix</italic> (Chrysomelidae: Cassidinae: Hispini) on <i><em full-name="Erythrina">Erythrina</italic></i>, Assam, India. (MP4 format, 3.41 min; film made and narrated by S. Ranade, 2020). https://binary.pensoft.net/file/531989Sachin Ranade, Kaniyarikkal Divakaran Prathapan, Hemant V. Ghate, Caroline S. Chaboo
